# Environmental and occupational respiratory diseases – 1044. Prevalence of recurrent wheezing in infants in a poor urban city in South Brazil

**DOI:** 10.1186/1939-4551-6-S1-P43

**Published:** 2013-04-23

**Authors:** Marilyn Urrutia Pereira, Juan Carlos Ivancevich, Dirceu Sole, Javier Mallol

**Affiliations:** 1 Pediatria, Santa CASA Uruguaiana, Brazil; 2 Cátedra De Inmunología, Universidad Del Salvador, Buenos Aires, Argentina; 3 Sao Paulo Federal University, Brazilian Society, Sao Paulo, Brazil; 4 Department of Pediatric and Respiratory Medicine, University of Santiago De Chile (USACH), Santiago, Chile

## Background

To identify the prevalence of recurrent wheezing in infants in the city of Uruguaiana, RS, Brazil.

## Methods

This is a cross-sectional study, part of the EISL (International Study of Wheezing in Infant). The parents or legal guardians of the infant aged 1-15 months attending health centers for immunization were interviewed and administered the EISL questionnaire, a standardized and validated instrument consisting of questions on demographic characteristics, wheezing, respiratory infections and risk factors during the period between January 2008 and July 2010.

## Results

Sampled infants (n=1061) had a mean age of 13.09 months with a prevalence of wheezing during their first year of life of 28.56%. 10,37% had 3-6 episodes. They lived in a poor area of the city, with low maternal education level (60,13% had ≥ 8 years of formal education) with an income < 500 US$ (99,81%). The exposure to prenatal maternal smoking was 9,61%, with 12.63% of maternal smoking and 34,31% of household smoking.The infant borned by caesarean section were 26,30% and a mean of breastfeeding of 3-4 months. Maternal history of asthma and rhinitis were 5,02% and 27,50% respectively.

## Conclusions

The prevalence of wheezing among infants living in a poor area of Uruguaiana is high. It is necessary to identify if the risk factors of wheezing in this low socio-economic level population differ from environmental stimuli found in developed countries.

